# N6-methyladenosine-mediated upregulation of MANF promotes ER stress resistance in renal cell carcinoma

**DOI:** 10.1038/s41419-025-07798-4

**Published:** 2025-07-03

**Authors:** Junjie Cen, Dongliang Zhao, Xin Shi, Jinlong Chen, Hang Zhou, Yanping Liang, Chengpeng Gui, Wei Chen, Junhang Luo, Xu Chen

**Affiliations:** 1 Department of Urology, The First Affiliated Hospital of Sun Yat-sen University, No. 58, Zhongshan Road II, Guangzhou, 510080 People’s Republic of China; 2 Department of Pathology, The First Affiliated Hospital of Sun Yat-sen University, No. 58, Zhongshan Road II, Guangzhou, 510080 People’s Republic of China; 3 Department of Pediatrics, The Third Affiliated Hospital of Sun Yat-sen University, No. 600, Tianhe Road, Guangzhou, 510630 People’s Republic of China; 4 Department of Laboratory Medicine, The First Affiliated Hospital of Sun Yat-sen University, No. 58, Zhongshan Road II, Guangzhou, 510080 People’s Republic of China

**Keywords:** Renal cell carcinoma, Cancer epidemiology

## Abstract

Renal cell carcinoma (RCC) remains a significant clinical challenge due to its increasing incidence and resistance to conventional therapy. This study identifies an unprecedented mechanism by which mesencephalic astrocyte-derived neurotrophic factor (MANF) contributes to RCC progression. From single-cell transcriptome, we observed a significant upregulation of MANF in RCC compared to its progenitor cells, the proximal tubular cells. Mechanistically, decreased N6-methyladenosine (m6A) modification on MANF mRNA, mediated by the upregulation of the m6A demethylase ALKBH5, led to reduced RNA degradation and increased MANF protein expression. Furthermore, while the common von Hippel-Lindau (VHL) deletion in RCC results in the accumulation of misfolded proteins and endoplasmic reticulum (ER) stress, increased MANF efficiently mitigated ER stress by binding to phosphorylated inositol-requiring enzyme-1 alpha (IRE1α) and inhibited its phosphorylation, indicating that MANF-mediated ER stress resistance compensates for the negative effects of VHL depletion and contributes to the survival of RCC. Our findings highlight a potential therapeutic strategy for RCC by targeting the m6A-mediated regulation, directly degrading MANF protein or inhibiting its function.

## Introduction

Renal cell carcinoma (RCC) is the most common kidney malignancy in adults, accounting for more than 90% of cases, and it affects over 400,000 individuals worldwide per year [1, 2]. RCC has several histopathological variants, among which approximately 80% cases belong to the clear cell RCC (ccRCC) subtype [3]. VHL gene mutation or loss is usually defined as the primary event in the pathogenesis of RCC. However, more detail mechanism driving progression and metastasis of RCC remains to be explored.

The endoplasmic reticulum (ER) is a quality-control organelle that plays a crucial role in regulating protein homeosis. When unfolded or misfolded proteins accumulate in ER, they activate a signaling mechanism called unfolded protein response (UPR) [4]. In spite of the fact that UPR provides cellular homeostasis by triggering pro-survival signaling cascades, chronic or severe activation of it results in apoptosis. Tumor cells rely on high levels of UPR to maintain rapid growth and metastasis. Cancer has great therapeutic potential due to the ability of the UPR to trigger pro-apoptotic mechanisms in response to irreversible cellular damage. Several studies with primary human ccRCC samples and retrospective analyses of ccRCC patients’ cohorts have revealed significant mitochondrial dysfunction and a decreased capacity for mitochondrial oxidation [5]. Loss of VHL in pre-cancerous kidney cells, which is considered as the hallmark of ccRCC tumorigenesis, induces ER stress and UPR responses leading to cell transformation and RCC induction [6]. Therefore, it is important to continue explore the precise regulatory mechanisms of UPR from pro-survival to pro-apoptosis in RCC.

MANF is an ER membrane-located protein, which was first identified in a murine ventral mesencephalic astrocyte cell line [7]. Studies have confirmed that MANF can perform many biological functions. For example, estrogen hormone therapy can attenuate myocarditis caused by immune checkpoint inhibitors through a MANF-dependent manner [8]. Also, MANF is also a therapeutic target of tubulointerstitial kidney disease [9]. Moreover, studies discover that MANF can exert suppressive effect in unresolved ER stress, possibly through IRE1α pathway [10, 11]. This effect has an important role in neural homeostasis, preventing neural cells from Parkinson’s disease changes by alleviating the ER stress caused by reactive oxygen species [12]. However, as today, the function of MANF remains unclear in RCC, as well as its relation with ER stress in RCC.

Post-transcriptional regulation of mRNAs is a crucial biological process, and N6-methyladenosine (m6A) is the most prevalent internal chemical modification of mRNAs in eukaryotes [13]. The N6-methylation of adenosine on mRNAs is a dynamic process involving the orchestration of writers, erasers, and readers [14]. In mammals, m6A methyltransferases [e.g., the methyltransferase-like (METTL) gene family] act as m6A writers, while fat mass- and obesity-associated protein (FTO) or alpha-ketoglutarate-dependent dioxygenase alkB homolog 5 (ALKBH5) primarily function as m6A erasers. In our analysis, MANF mRNA had abundant m6a sites, and was found to be regulated by ALKBH5.

In the present study, MANF was identified to be overexpressed in renal cancer cells through single-cell sequencing analysis. It correlated with RCC aggressiveness and contributed to enhanced tumor cell proliferation and invasiveness. Mechanism study confirmed that MANF was also a crucial regulator of ER stress balance in RCC cells. This regulation is particularly significant given the context of von Hippel-Lindau (VHL) status, which is frequently altered in RCC. In VHL-deficient RCC cells, MANF’s protective effects against cellular stress were even more pronounced. Moreover, ALKBH5 reduced the m6A modification at a 3’UTR site of MANF mRNA, leading to increased mRNA levels and enhanced stability through interaction with YTHDF proteins. Through these findings, a novel epistatic mechanism was revealed in which MANF expression was epigenetically regulated via m6A modification by ALKBH5, thereby promoting a pro-survival unfolded protein response (UPR) in RCC cells. This m6A-mediated regulation of MANF underscored its potential as a therapeutic target in RCC.

## Materials and Methods

### Clinical samples and patient follow-up data

The protocol regarding collection of patient information and usage of pathological samples were approved by the Ethical Committees of the First Affiliated Hospital of Sun Yat-sen University (Guangzhou, China), which declares Helsinki Declaration agreement. Informed consent was obtained from all patients. The present cohort included 192 ccRCC patients, in which the surgery time ranged from December 1999 to December 2015, with a median follow-up time of 103.5 months. Overall survival (OS) was defined as the time interval from surgery to death, while recurrence-free survival (RFS) was defined as the time interval from surgery to first notice of ccRCC recurrence. Formalin-fixed and paraffin-embedded (FFPE) samples were retrieved for RNA extraction (ThermoFisher, MA, USA) and immunohistochemistry (IHC) staining. In the Chi-square, Kaplan-Meier survival, and Cox analyses, the median RNA expression of ALKBH5 was used as a cut-off value for separating the high and low ALKBH5 groups. The Cancer Genome Atlas (TCGA) ccRCC data source was downloaded from the Firebrowse website (http://www.firebrowse.org/).

### Bioinformatics analysis

Differential analysis of genes in RNA-seq and heatmap generation were performed using the tidyverse and pheatmap packages in R software (version 4.1.3, https://www.r-project.org/), respectively. m6A site prediction was performed using the SRAMP online algorithm (http://www.cuilab.cn/sramp).

For public scRNA-seq data, we collected scRNA-seq count matrix of 195 samples described in 10 datasets (GSE159115, GSE131685, GSE224630, GSE207493, PMC8138872, phs002065, Mendeley, PMC6104812, SRZ190804) were downloaded and used. Raw gene expression matrices were imported and processed using the Seurat R package (version 3.1.5). Low-quality cells were removed as previous study. And 195 samples were then merged, and batch effects were minimized using Harmony and RPCA R package (version 1.0). Gene expression matrices of the remaining high-quality cells were normalized to the total cellular UMI counts. The normalized expression was scaled (scale. factor =10,000) by regressing out the total cellular UMI counts and percentage of mitochondrial gene. Highly variable genes (top 2000) were extracted using the Seurat FindVariableGenes function. Then, we performed principal component analysis (PCA) analysis using high variable genes, and significant PCs (top 50) were selected to perform dimension reduction. Clusters were found using FindClusters function (dims.use = 1:30, resolution = 1). The RunUMAP analysis was used for dimension reduction and visualization of gene expression. The cell lineage trajectory of Treg was inferred using the Monocle2 R package. We used the “differentialGeneTest” function to derive DEGs from each cluster and genes with a q-value < 0.01 were used to order the cells in pseudotime analysis.

### Cell culture

In the present study, a normal kidney cell line (293) and three RCC cell lines (786-O, ACHN and Caki-1) were used [American Type Culture Collection (ATCC)]. Roswell Park Memorial Institute (RPMI) 1640 medium (Gibco, NY, USA) was used to culture 786-O and ACHN cells, and Dulbecco’s Modified Eagle Medium (Gibco) was used to culture 293 and Caki-1cells. All media were supplemented with 10% fetal bovine serum (FBS, ExCell Bio, Shanghai, China), and the cells were cultured at 37°C in 5% CO_2_ in a bio-incubator (ThermoFisher). Short tandem repeat (STR) DNA genotype analysis was performed to ensure the authenticity of cell line origins. Mycoplasma infection was periodically checked with a kit (Beyotime, Shanghai, China).

### Small interfering RNA (siRNA) transfection

siRNAs were applied to manipulate certain gene expression. siRNAs were synthesized by RiboBio (Guangdong, China), and JetPEI reagent (Polyplus-transfection, Illkirch, France) was used for siRNA transfection.

### CRISPR/Cas9 method

VHL and ALKBH5 knockout (KO) RCC cell lines were generated using the CRISPR/Cas9 method. Each knockout gene was attempted with four sgRNA sequences, and the clones with best knockout efficiency were selected for subsequent experiments. In brief, PlentiCRISPR V2 plasmids containing different sgRNAs were transfected into HEK-293T cells, along with the lentiviral psPAX2 packing plasmid and pMD2.G envelope plasmid. Medium was changed after 12 h of transfection. After 48 h, the supernatants containing the lentivirus were collected, centrifuged, and filtered with a 0.45μm membrane. To generate stable RCC KO cell lines, cells were infected with the lentiviruses followed by 1 μg/ml puromycin (Beyotime) selection for a least 1 week. The sgRNA sequences are listed in Table S1.

### Quantitative real-time PCR (qRT-PCR) analysis

Total RNA was extracted and purified with TRIzol reagent (ThermoFisher) following the manufacturer’s protocol, and the yield was measured using a NanoDrop (ThermoFisher). For cDNA synthesis, 1 μg of total RNA and an iScript cDNA Synthesis Kit (Bio-Rad, CA, USA) were used. After amplification of cDNA, the product was mixed with specific primers and 2X SYBR Green Pro Taq HS Premix II (AGbio, Hunan, China), and gene expression was analyzed using a QuantStudio 5 real-time PCR instrument (ThermoFisher). Glyceraldehyde 3-phosphate dehydrogenase (GAPDH) was used as an internal normalization control for mRNA, and the 2^-ΔΔCt^ method was used to calculate the relative expression of different genes. The qRT-PCR primers are listed in Table S1.

### Western blot analysis

Cells were collected and lysed with ice-cold radioimmunoprecipitation assay (RIPA) buffer (Beyotime) supplemented with proteinase inhibitor (Beyotime). The lysate was centrifuged at 13,000 rpm for 15 min, and the supernatant was collected. A bicinchoninic acid assay (ThermoFisher) was used to determine the protein concentration of each sample. The sample proteins were loaded into sodium dodecyl sulfate polyacrylamide electrophoresis (SDS-PAGE) gels, and electrophoresis was performed. After separation, proteins were transferred to polyvinylidene fluoride membranes (Roche, USA). After blocking with non-fat milk and washing with phosphate-buffered saline (PBS), the membranes were incubated with specific primary antibodies at 4°C overnight. After washing with PBS, the membranes were incubated with secondary anti-mouse/rabbit IgG antibodies, and SuperSignal West Pico PLUS Chemiluminescent Substrate (ThermoFisher) was used for exposure on a FluorChem E System (General Electric, USA). The following antibodies were used in the present study: VHL (#68547, Cell Signaling Technology, USA), HIF1a (D1S7W, Cell Signaling Technology), ALKBH5 (10H24L9, ThermoFisher), MANF (10869-1-AP, ProteinTech, China), BiP (C50B12, Cell Signaling Technology), IRE1α (27528-1-AP, ProteinTech), p-IRE1α (PA1-16927, ThermoFisher) and GAPDH (D16H11, Signaling Technology).

### Colony-forming assay

For evaluation of proliferation ability, a colony-forming assay was performed. After treatments, cells (500 cells/well) were seeded into 6-well plates, and medium was changed periodically. After 1 week of incubation, cell colonies were fixed with 4% formaldehyde (Biosharp, Anhui, China) and stained with 0.4% crystal violet (Biosharp). The colonies were counted, and different groups were compared.

### CCK-8 assay

For evaluation of proliferation ability, a CCK-8 assay was performed. After treatments, cells (1000 cells/well) were seeded into 96-well plates. After certain incubation time and the cells were attached, 10 μl of CCK-8 reagent (Biosharp) was added to each well and incubated for 1 h. The reaction product of CCK-8 was measured with a Varioskan LUX machine (ThermoFisher) at a wavelength of 450 nm. Data was normalized relatively to day 0 or no treatment group. Each experiment condition had at least 3 repeated wells, and each experiment had three parallel tests.

### Wound-healing assay

Wound-healing assay was performed to assess the migratory ability of RCC cell lines. Cells were seeded into 6-well plates at a high density to reach 100% confluency. After cell adhesion, cells were washed with PBS to remove floating cells. A wounded area located at the central diameter was then created using a 1 ml pipette tip. The floating cells were again washed out with PBS, and fresh medium with 10% FBS was added to each well. Images at 0 h and 24 h were captured with an IX83 inverted microscope (Olympus, Tokyo, Japan), and the relative migrative distance (the occupied width at 24 h relative to 0 h) was calculated. Finally, data was normalized relatively to no treatment group.

### Transwell assay

Transwell assays were performed to evaluate migration and invasion abilities. Cells treated with different conditions were adjusted to a total cell number of 10,000 in serum-free medium and seeded into a Transwell insert (Corning, NY, USA). For migration assay, the inserts were directly placed into the wells containing medium with 10% FBS as a chemo-attractant. For the invasion Transwell assay, the insert was first coated with a 100 μl layer of 2% Matrigel (Corning) overnight at 37 °C, and the same cell number was seeded. The inserts were then also placed into a 24-well plate with medium containing 10% FBS. After incubation for 8 h (for migration) or 24 h (for invasion), the inserts were collected, fixed with 4% formaldehyde (Biosharp) for 1 h, and stained with 0.4% crystal violet (Biosharp) 15 min. Cells in the upper layer of the insert were removed with a cotton swab. The cells remaining on the lower surface of the insert were visualized using an IX83 inverted microscope (Olympus), and images of five random high-power fields were acquired. The cell number of each image was counted, and the median cell number of all five images was calculated. Then the relatively migrated/invaded cells were normalized to 0 h and no treatment group.

### m6A pull-down assay

m6A pull-down was performed with a Magna MeRIP m6A Kit (Merck KgaA, Darmstadt, Germany) following the manufacturer’s protocol. Briefly, total RNA was first fragmentated with fragmentation buffer and heated to 94 °C using a thermocycler. If whole length of RNA was needed, fragmentation was skipped. Magnetic A/G beads were washed with immunoprecipitation (IP) buffer, and anti-m6A and control mouse IgG antibodies were added to the beads followed by incubation. RNA was added to the antibody-bound beads, along with RNase inhibitor and IP buffer. After an incubation time of 2 h at 4 °C, RNA-conjugated beads were eluted, and the final product was evaluated using qRT-PCR with specific primers (Table S1).

### RNA immunoprecipitation (RIP)

To perform the RIP assay, cells were crosslinked in dishes with 0.75% formaldehyde (Biosharp). Cells were then collected, centrifuged, and resuspended in RIP buffer. Cells were sheared with a Dounce homogenizer and centrifuged. The target protein and control IgG antibodies were added to the cell lysate, and protein A/G magnetic beads (Abcam, Cambridge, UK) were later added to the mixture. Following incubation and washing, the RNA in the mixture was purified using TRIzol reagent and subjected to qRT-PCR analysis.

### Luciferase reporter assay

In brief, different full-length and truncated genes were inserted into the psiCHECK-2 vector. 293 T cells (3 × 10^3^ cells/well) were seeded into 96-well plates. After cell attachment, 50 ng of the psiCHECK-2 vector and 5 ng of the Renilla vector (pRL-TK) were used for transfection of cells in each well. After 48 h of incubation, the luciferase activity of each well was determined with a Dual Luciferase Reporter Assay Kit (Promega, WI, USA) on a Varioskan LUX machine (ThermoFisher, MA, USA). The luciferase activity was normalized to the Renilla control.

### Actinomycin D assays

An actinomycin D assay was used to determine RNA stability. Cells were treated with or without 2 μg/ml actinomycin D (Aladdin, China) to halt RNA synthesis. Thereafter, the remaining RNA in the cells was extracted with a RNeasy MinElute Cleaning Kit (Qiagen, Germany) and subjected to qRT-PCR analysis.

### Transmission electron microscopy (TEM)

Cells or tissues were fixed with a 0.1 M phosphate buffer containing 2.5% glutaraldehyde. After the fixation process, the sample was washed with 0.1 M phosphate buffer for 3 times, and post-fixed with 1% buffered osmium for 2 h. After post-fixation, the sample was again washed with 0.1 M phosphate buffer for 3 times. Then, a series of gradient ethanol was used to dehydrate the sample (30%, 50%, 75%, 95%, and 100% ×3, each gradient for 15 min). Thereafter, the sample was embedded overnight. Ultrathin sections were cut with a Leica EM UC7 Ultracut microtome (Leica, IL, USA). After a final step of uranyl acetate/lead citrate staining and 0.1 M phosphate buffer washing, images of the samples were acquired using a JEM-1200EX electron microscope (JEOL, Tokyo, Japan).

### Immunofluorescence (IF) assay

For IF assays, cells were seeded onto 15-mm glass-bottom plates (Nest, China). After the cells attached to the glass plates, they were rinsed with PBS for 3 times and fixed with 4% polyformaldehyde (Biosharp) for 30 min. After permeabilizing with 0.5% Triton X-100/PBS for 15 min and blocking with 5% bovine serum albumin (Beyotime) for 1 h, the primary antibodies were added to the plated and incubated overnight at 4 °C. Then, after washing with PBS, fluorescent secondary antibodies were added to incubation at room temperature for 1 h. The nuclei were stained with 4’,6-diamidino-2-phenylindole (DAPI) for 5 min. Fluorescence images were captured on an IX83 inverted microscope (Olympus). Antibodies used in IF are as followed: MANF (10869-1-AP, ProteinTech, China) and IRE1α (sc-390960, Santa Cruz, USA).

### Animal experiments

All protocols regarding animal experiments and manipulations were designed in accordance to the Guide for the Care and Use of Laboratory Animals (National Research Council, USA), and they were approved by the Institutional Animal Care and Use Committee of Sun Yat-sen University, which follows the rules of Basel Declaration. Animal number was estimated to ensure adequate statistical power. Three-week-old nude BALB/c male mice were purchased from GemPharmatech (Guangdong, China) and randomized to two groups, which was blind to the investigators. Stable knockout of ALKBH5 in RCC cells was achieved by lentiviral transfection. For the tail vein injection lung metastasis model, ALKBH5 knockout and control cells were also transfected with luciferase, and successful transfection was confirmed by adding the luciferin substrate to cell lysate and detecting the illumination. A total of 2 × 10^6^ cells in 200 μl of PBS were injected into the circulation of each mouse through the tail vein. Mice were monitored every 2 weeks using an IVIS Spectrum In Vivo Imaging System (PerkinElmer, MA, USA) to evaluate the growth of experimental metastases. Before imaging the mice, luciferin substrate was injected into the abdomen and circulated for 15 min. After 8 weeks, the mice were euthanized, and lungs were dissected, collected, fixed in 4% formaldehyde (Biosharp), and subjected to HE staining. Pulmonary metastatic nodules were inspected using an IX83 inverted microscope (Olympus, Tokyo, Japan).

For the subcutaneous tumor formation model, 5 × 10^6^ cells were injected subcutaneously into the left axillary area of each mouse, and the long and short axes of tumor size was measured every week. The tumor volume was calculated using the following formular: tumor volume = 0.5 × long axis × short axis × short axis. After 4 to 6 weeks, the mice were euthanized, and subcutaneous tumors were dissected, collected, and fixed in 4% formaldehyde (Biosharp). The tumors were subjected to HE and IHC staining.

### Statistical analysis

GraphPad Prism 9 was utilized to perform statistical analyses and plot graphs. Sample size was estimated to ensure adequate statistical power. Student’s t-test was used to compare differences between two groups. Analysis of variance (ANOVA) was used to compare three or more groups, along with Tukey’s multiple comparisons between groups. Pearson’s analysis was used to evaluate correlations between two groups. Clinical parameters between high and low ALKBH5 groups were analyzed with a Chi-square test. The OS and RFS between the high and low ALKBH5 groups were analyzed with Kaplan-Meier curves (log-rank test), univariate Cox analysis, and multivariate Cox analysis. All experiments were performed three times. Quantitative data are presented as the mean ± standard deviation. All P values < 0.05 were considered significant (P < 0.05 for *, P < 0.01 for **, and P < 0.001 for ***).

## Results

### MANF loss triggers ER stress in RCC cells

**Fig. 1:**
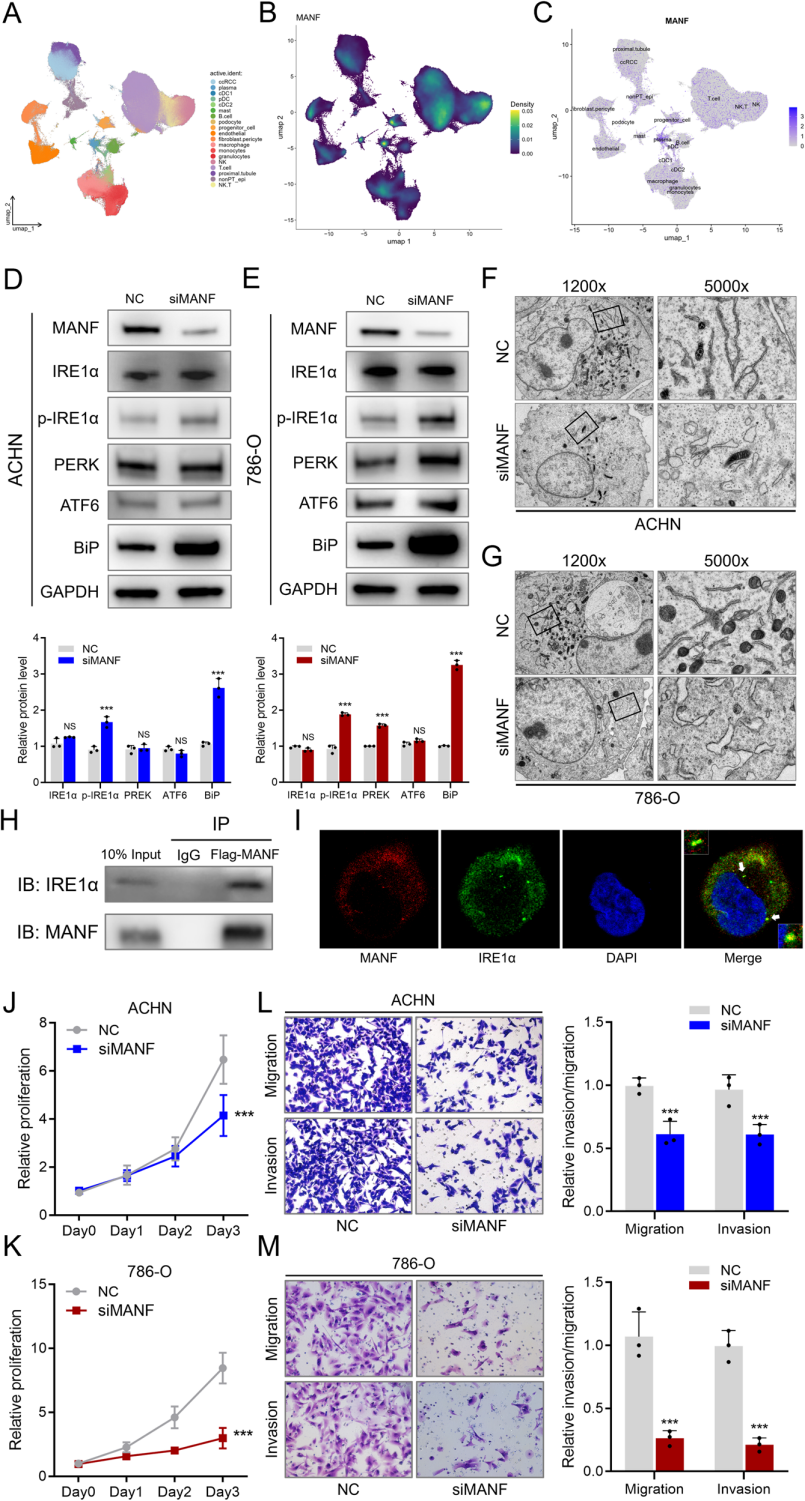
MANF loss triggers ER stress in RCC cells. **A** Cell population of single cell sequencing data (RCC vs. normal adjacent kidney tissue). **B** MANF expression of single cell sequencing data. **C** MANF expression in different cell population of single cell sequencing data. **D** Upper: western blot detecting MANF and the ER stress effector proteins in ACHN cell line with NC or siMANF. Lower: quantification of the ER stress effectors in the western blot experiment. **E** Upper: western blot detecting MANF and the ER stress effector proteins in 786-O cell line with NC or siMANF. Lower: quantification of the ER stress effectors in the western blot experiment. **F** Representative TEM images of ER changes in ACHN cell line with NC or ALKBH5 KO. High power fields showing detailed structure of ER. **G** Representative TEM images of ER changes in 786-O cell line with NC or ALKBH5 KO. High power fields showing detailed structure of ER. **H** Immunoprecipitation assay assessing the interaction between IRE1α and MANF in 293 T cells. IgG was used as negative control. **I** Immunofluorescence assay showing the intracellular location of MANF and IRE1α. The nuclei were stained with DAPI. White arrows indicated co-localization of MANF and IRE1α. **J** CCK8 assay of NC and siMANF in ACHN cell line. Proliferation rates were normalized to day 0. **K** CCK8 assay of NC and siMANF in 786-O cell line. Proliferation rates were normalized to day 0. **L** Transwell assay (migration and invasion) of NC and siMANF in ACHN cell line. Representative pictures shown in left and quantification data shown in right. **M** Transwell assay (migration and invasion) of NC and siMANF in 786-O cell line. Representative pictures shown in left and quantification data shown in right.

Our pre-experiment discovered that MANF might have critical regulatory function in RCC. The MANF gene was originally identified in the rat ventral mesencephalic astrocyte cell line 1 (VMCL1) [7]. MANF is widely expressed in the central nervous system and is considered to be involved in neural development [15]. Recently, it has been reported that MANF protects developing fetal neurons from ER stress damage induced by ethanol exposure [16]. Thus, we also investigated whether MANF has an ER stress-protective role in RCC cells. ER stress and the resultant unfolded protein response (UPR) are primarily regulated by three ER-localized transmembrane proteins, namely, inositol-requiring kinase (IRE1α), protein kinase RNA-like endoplasmic reticulum kinase (PERK), and activating transcription factor 6 (ATF6) [17]. Single cell sequencing date was utilized to explorer the role of MANF in RCC. Result showed that MANF was overexpressed in ccRCC group, compared to the normal proximal tubule group (Fig. 1A–C). After knockdown of MANF, phosphor-IRE1α demonstrated the most significant change among the three UPR effectors (Fig. 1D, E). In addition, BiP, the hallmark of UPR, was significantly increased, indicating the escalation of ER stress (Fig. 1D, E). Further, TEM analysis revealed classic subcellular structure changes in response to ER stress, including dilation and swelling of the ER (Fig. 1F, G). Immunoprecipitation confirmed the interaction of IRE1α and MANF (Fig. 1H), and immunofluorescence showed the co-localization of IRE1α and MANF (Fig. 1I). After knockdown of MANF, the excruciating ER stress status of RCC cells impeded their proliferation and invasiveness abilities (Fig. 1J–M). These results indicated that MANF stabilized the ER status in RCC cells, promoting normal function of RCC cells, and loss of MANF triggered significant ER stress *via* the IRE1α pathway.

### ALKBH5 decreases the m6A abundance on MANF mRNA 3’-UTR

**Fig. 2:**
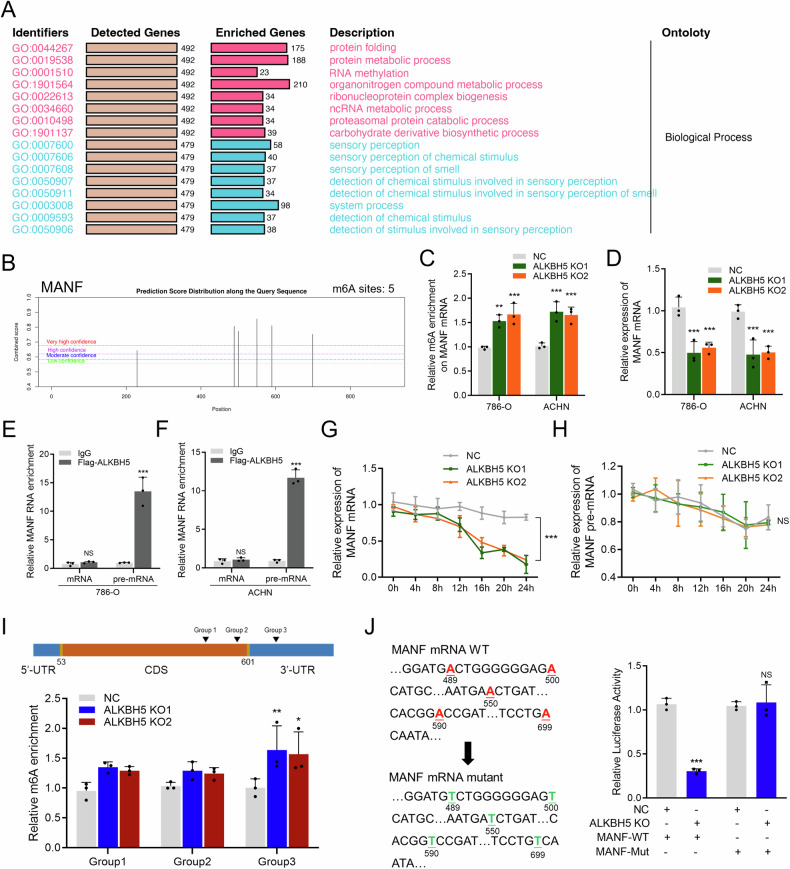
ALKBH5 decreases the m6A abundance on MANF mRNA 3’-UTR. **A** Pathway analysis of high/low MANF group of single cell sequencing data. **B** m6A sites predicted by SRAMP m6A prediction algorithm, showing 5 sites with high prediction confidence. **C** m6A pull-down assay assessing the m6A abundance of MANF in ACHN and 786-O cell lines with NC or ALKBH5 KO. Enrichment levels were normalized to NC group. **D** qRT-PCR detection of MANF expression level in in ACHN and 786-O cell lines with NC or ALKBH5 KO. Enrichment levels were normalized to NC group. **E** RIP assay assessing the binding of ALKBH5 on MANF mRNA and pre-mRNA in 786-O cell line. Enrichment levels were normalized to IgG, mRNA group. **F** RIP assay assessing the binding of ALKBH5 on MANF mRNA and pre-mRNA in ACHN cell line. Enrichment levels were normalized to IgG, mRNA group. **G** Actinomycin D assay assessing the stability of MANF mRNA in 786-O cell lines with NC or ALKBH5 KO. Levels were normalized to NC group, 0 h. **H** Actinomycin D assay assessing the stability of MANF mRNA in ACHN cell lines with NC or ALKBH5 KO. Levels were normalized to NC group, 0 h. **I** Upper: schematic diagram of MANF mRNA structure and the locations of three groups of m6A sites. Lower: m6A pull-down assay assessing three groups of m6A abundance of MANF in 786-O cell lines with NC or ALKBH5 KO. Enrichment levels were normalized to NC group. **J** Luciferase reporter assay of MANF point mutation in 293 cell lines with NC or ALKBH5 KO. Left: schematic diagram of MANF point mutation. Right: quantification of the Luciferase reporter assay. Levels were normalized to NC/MANF-WT group.

We further explored the reason why MANF was overexpressed in RCC. Pathway analysis showed that RNA methylation was associated with MANF (Fig. 2A). Also, MANF was predicted to have abundant m6A sites as predicted by SRAMP m6A algorithm [18] (Fig. 2B). We wondered if m6A sites could regulate the expression of MANF. After literature review, we discovered that RNA demethylase ALKBH5 had important function. The relation between ALKBH5 and MANF was further tested. Methylated RNA immunoprecipitation (MeRIP) and qRT-PCR confirmed that ALKBH5 bound to MANF pre-mRNA and decreased its m6A abundance, and MANF mRNA (but not pre-mRNA) expression was decreased after knockout of ALKBH5 (Fig. 2C–F and Figure S1A, B). This alteration resulted from a decreased MANF mRNA stability, as MANF mRNA half-life significantly diminished (Fig. 2G, H).

The SRAMP algorithm predicted five m6A sites on MANF mRNA. Site-specific primers were designed to further elucidate at which site(s) the m6A levels were altered after ALKBH5 knockout. Due to the proximity of their locations, the five m6A sites were divided into three groups as follows: group 1, 489nt and 500nt; group 2, 550nt and 590nt; and group 3, 699nt (Fig. 2I). MeRIP and qRT-PCR demonstrated that ALKBH5 knockout significantly increased the m6A level in group 3 (Fig. 2I). A luciferase reporter assay using site-specific mutations further confirmed that group 3 was responsible for the mRNA unstability. Of note, group 3 was located on the 3’-UTR of MANF mRNA (Fig. 2J and Figure S1C). These results indicated that ALKBH5 decreases the m6A abundance on MANF mRNA 3’-UTR, thus ensuring MANF mRNA stability.

### ALKBH5 is upregulated in RCC tissues and correlated with RCC patient prognosis

**Fig. 3:**
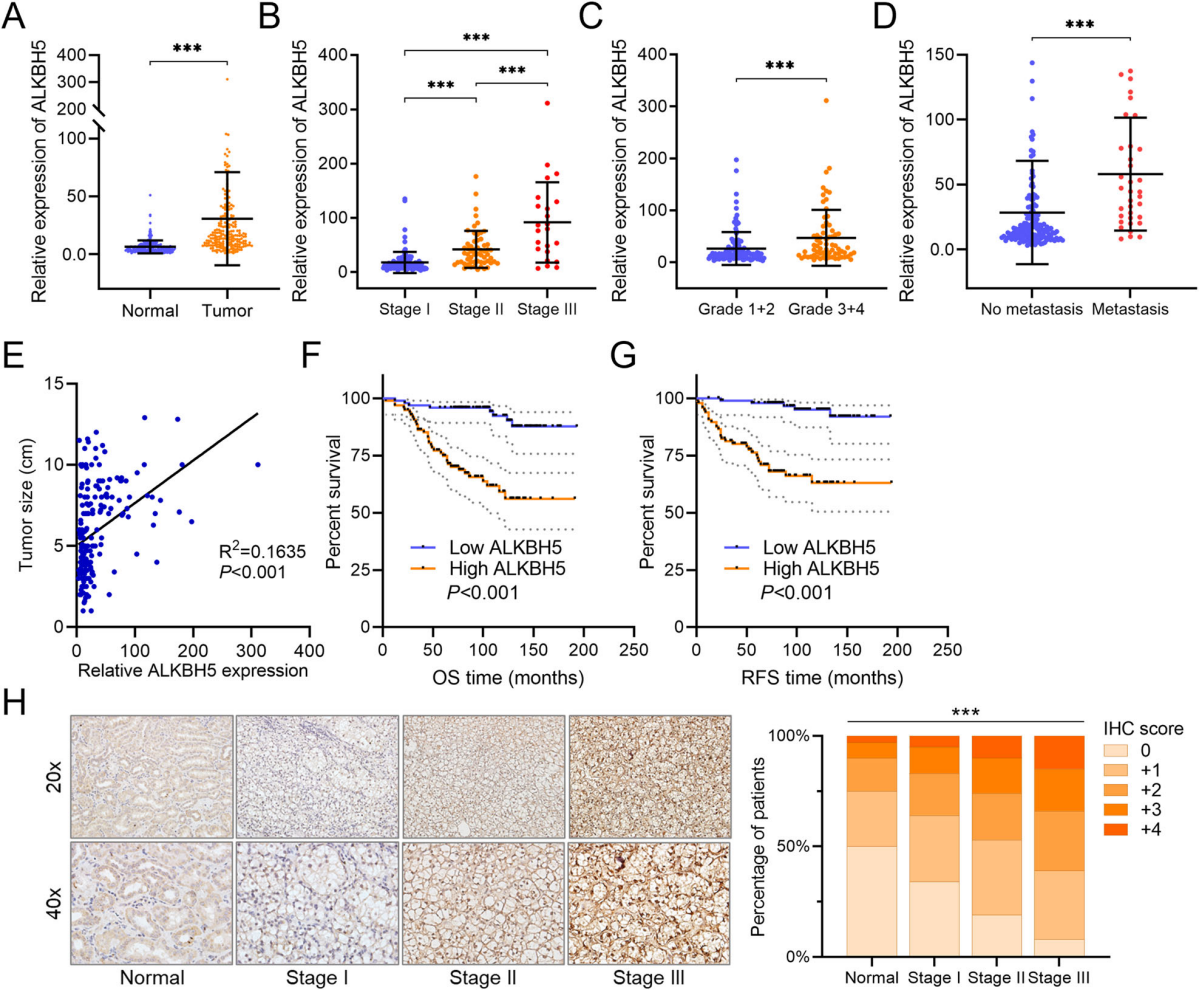
ALKBH5 is upregulated in RCC tissues and correlated with RCC patient prognosis. **A** The relative expression of ALKBH5 RNA in tumors and adjacent normal kidney tissues of 192 RCC patients. The expression was quantified by qRT-PCR. **B** The relative expression of ALKBH5 RNA in patients with different clinical stages of 192 RCC patients. The expression was quantified by qRT-PCR. **C** The relative expression of ALKBH5 RNA in patients with different ISUP grades of 192 RCC patients. The expression was quantified by qRT-PCR. **D** The relative expression of ALKBH5 RNA in tumors of RCC patients who did or did not eventually develop distant metastasis. The expression was quantified by qRT-PCR. **E** The correlation analysis between ALKBH5 RNA expression and tumor size in 192 RCC patients. **F** OS survival analysis of high/low ALKBH5 expression group in 192 RCC patients. The median ALKBH5 expression was used as cut-off point. **G** RFS survival analysis of high/low ALKBH5 expression group in 192 RCC patients. The median ALKBH5 expression was used as cut-off point. **H** Representative pictures and quantification of ALKBH5 IHC staining in normal and different clinical stages of the192 RCC patients.

For further validation, clinical information and pathological specimens were obtained from a cohort of 192 ccRCC patients from First Affiliated Hospital of Sun Yat-sen University. Compared to noncancerous kidney tissue, the expression of ALKBH5 was significantly elevated in tumors (Fig. 3A). Moreover, higher expression of ALKBH5 was correlated with advanced clinical stages, International Society of Urological Pathology (ISUP) grades, metastasis and larger tumor size (Fig. 3B–E, Table 1). Survival analysis demonstrated that patients with higher ALKBH5 expression had poorer OS and RFS (Fig. 3F, G), and Cox analysis further confirmed ALKBH5 was an independent prognostic factor for the survival and recurrence of ccRCC patients (Tables 2 and 3). Consistent with RNA expression, the protein level of ALKBH5 was also elevated in advanced clinical stages (Fig. 3H). These data demonstrated that ALKBH5, functioning as an oncogene, has important clinical implications in ccRCC patients.

**Table 1:** Association of ALKBH5 expression with clinicopathological characteristics in 192 ccRCC patients.

Parameter	Total	ALKBH5 expression		*p* value
		High	Low	
**Age(y)**				
< 60	131	60	71	0.121
≥ 60	61	36	25	
**Gender**				
Female	65	35	30	0.542
Male	127	61	66	
**Size**				
< 4 cm	65	18	47	<0.001
≥ 4 cm	127	78	49	
**T stage**				
T1	111	31	80	<0.001
T2	58	45	13	
T3	23	20	3	
**Clinical (TNM) stage**				
I	108	30	78	<0.001
II	58	43	15	
III	26	23	3	
**ISUP grade**				
1 + 2	123	51	72	0.003
3 + 4	69	45	24	
**Surgery type**				
Open surgery	121	71	50	0.002
Laparoscopic surgery	71	25	46

**Table 2:** Univariate and multivariate Cox regression analyses of different parameters on overall survival.

Parameter	Univariate Analysis		Multivariate Analysis	
	HR (95%CI)	*P* Value	HR (95%CI)	*P* Value
Age	1.039 (1.014-1.064)	0.002	1.028 (1.002–1.054)	0.036
Gender (Female vs. male)	0.946 (0.503-1.778)	0.863	—	—
T stage (T2-T3 vs. T1)	2.115 (1.353-3.307)	0.001	0.819 (0.216–3.110)	0.770
Clinical (TNM) stage (II–III vs. I)	2.179 (1.383-3.431)	<0.001	1.104 (0.285–4.275)	0.886
ISUP (3 + 4 vs. 1 + 2)	1.822 (1.186-2.801)	0.006	1.535 (0.965–2.441)	0.070
Surgery type (Lapar. vs. open)	0.463 (0.261-0.823)	0.009	0.584 (0.316–1.078)	0.085
ALKBH5 expression (High vs. low)	3.516 (2.033-6.082)	<0.001	2.796 (1.481–5.277)	0.002

*HR* hazard ratio, *CI* confidence interval.

**Table 3:** Univariate and multivariate Cox regression analyses of different parameters on recurrent-free survival.

Parameter	Univariate Analysis		Multivariate Analysis	
	HR (95%CI)	*P* Value	HR (95%CI)	*P* Value
Age	1.028 (1.002–1.054)	0.035	1.016 (0.989–1.043)	0.257
Gender (Female vs. male)	1.090 (0.540–2.186)	0.807	—	—
T stage (T2-T3 vs. T1)	2.096 (1.294–3.395)	0.003	1.501 (0.213–10.566)	0.683
Clinical (TNM) stage (II-III vs. I)	2.000 (1.235–3.240)	0.005	0.523 (0.073–3.721)	0.517
ISUP (3 + 4 vs. 1 + 2)	2.483 (1.531–4.027)	<0.001	2.108 (1.264–3.517)	0.004
Surgery type (Lapar. vs. open)	0.659 (0.386–1.125)	0.126	—	—
ALKBH5 expression (High vs. low)	4.850 (2.464–9.548)	<0.001	4.610 (2.160–9.842)	<0.001

HR=hazard ratio. CI= confidence interval.

### ALKBH5 promotes proliferation and invasiveness of RCC cells

**Fig. 4:**
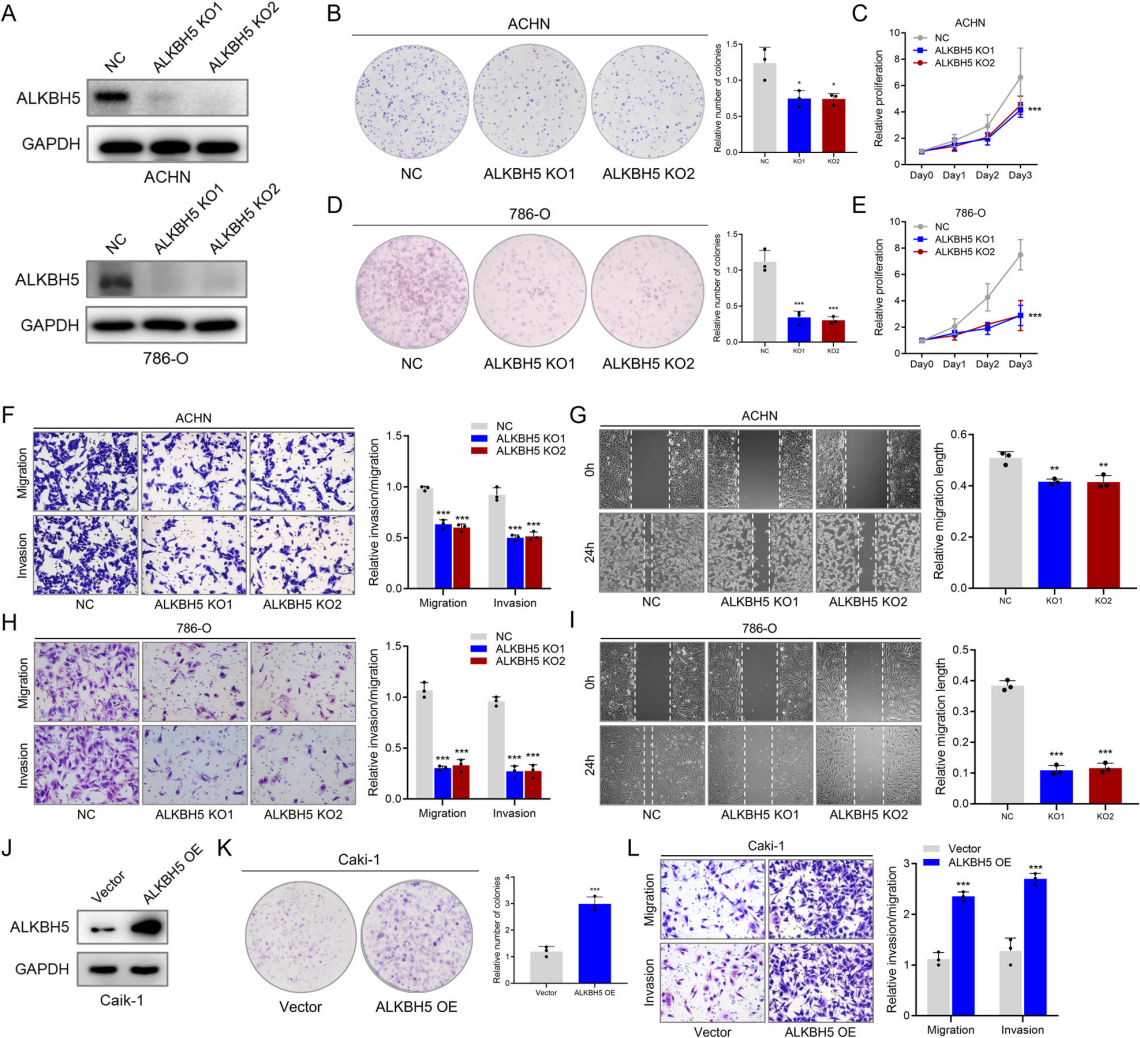
ALKBH5 promotes proliferation and invasiveness of RCC cells. **A** Western blot validation of ALKBH5 knockout in two RCC cell line, ACHN (upper) and 786-O (lower). **B** Colony forming assay of NC, ALKBH5 KO1 and ALKBH5 KO2 in ACHN cell line. Representative pictures shown in left and quantification data shown in right. **C** CCK8 assay of NC, ALKBH5 KO1 and ALKBH5 KO2 in ACHN cell line. Proliferation rates were normalized to day 0. **D** Colony forming assay of NC, ALKBH5 KO1 and ALKBH5 KO2 in 786-O cell line. Representative pictures shown in left and quantification data shown in right. **E** CCK8 assay of NC, ALKBH5 KO1 and ALKBH5 KO2 in 786-O cell line. Proliferation rates were normalized to day 0. **F** Transwell assay (migration and invasion) of NC, ALKBH5 KO1 and ALKBH5 KO2 in ACHN cell line. Representative pictures shown in left and quantification data shown in right. **G** Wound heal assay of NC, ALKBH5 KO1 and ALKBH5 KO2 in ACHN cell line. Representative pictures shown in left and quantification data shown in right. **H** Transwell assay (migration and invasion) of NC, ALKBH5 KO1 and ALKBH5 KO2 in 786-O cell line. Representative pictures shown in left and quantification data shown in right. **I** Wound heal assay of NC, ALKBH5 KO1 and ALKBH5 KO2 in 786-O cell line. Representative pictures shown in left and quantification data shown in right. **J** Western blot validation of ALKBH5 overexpression in Caki-1 cell line. **K** Colony forming assay of Vector and ALKBH5 OE in Caki-1 cell line. Representative pictures shown in left and quantification data shown in right. **L** Transwell assay (migration and invasion) of Vector and ALKBH5 OE in Caki-1 cell line. Representative pictures shown in left and quantification data shown in right.

To further explore the influence of ALKBH5 on RCC cells, two ALKBH5 KO clones were constructed, and their KO efficiency was confirmed (Fig. 4A). Knockout of ALKBH5 significantly increased the m6A level in RCC cells, which was consistent with its demethylase function (Figure S2A). Compared to the control group, ALKBH5-deficient RCC cells showed decreased colony-forming ability and proliferation (Fig. 4B–E). Also, as ALKBH5 expression decreased, the migration and invasion abilities of RCC cells were also significantly reduced (Fig. 4F–I). In contrast, when ALKBH5 was overexpressed in RCC cells (Fig. 4J), m6A level was significantly lower (Figure S2B), and the proliferation and aggressiveness of RCC cells decreased (Fig. 4K, L). These results showed that ALKBH5 acts as an oncogene in RCC, to promote proliferation and invasiveness of RCC cells.

### YTHDF2 down-regulates the stability of MANF mRNA in an m6A-dependent manner

**Fig. 5:**
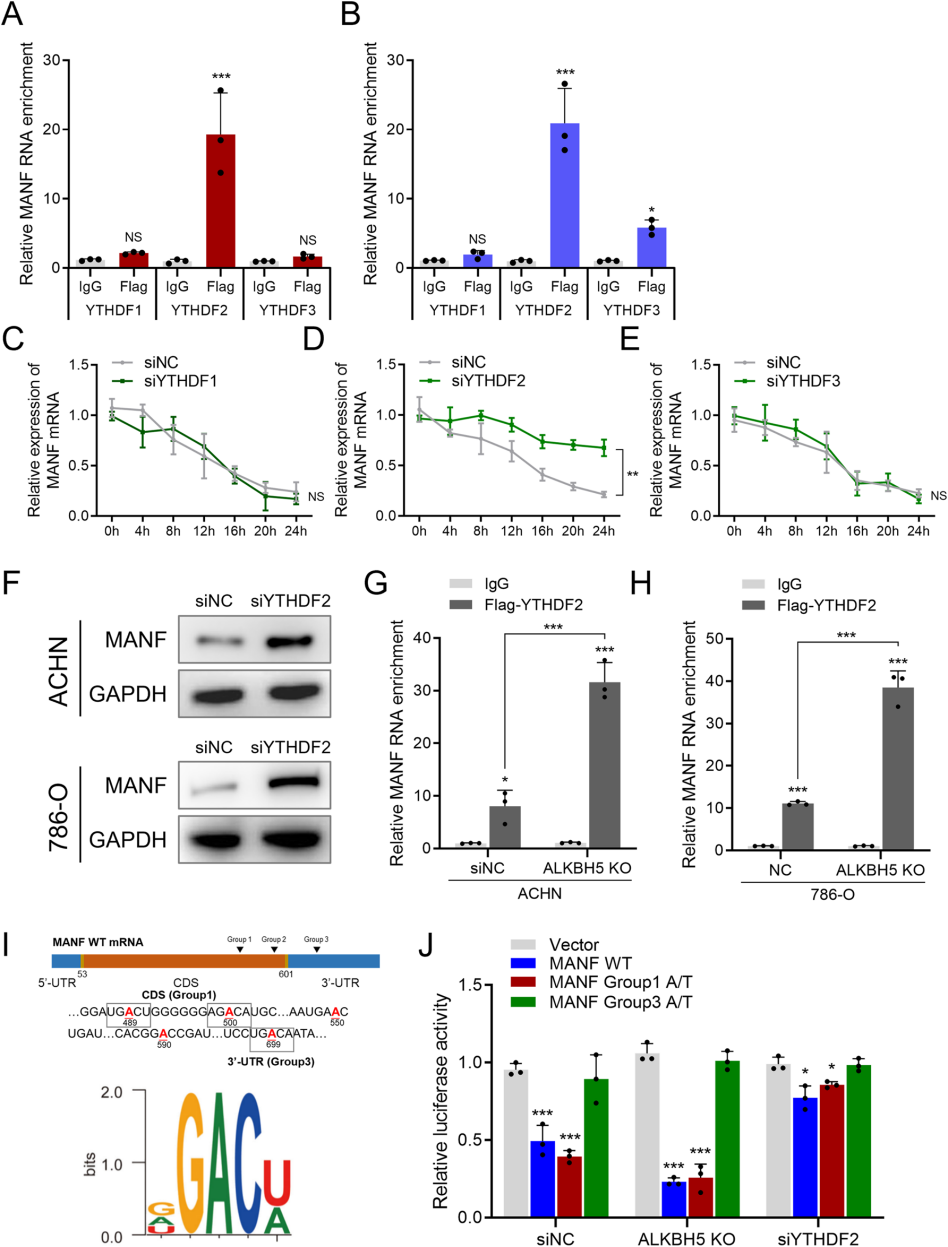
YTHDF2 down-regulates the stability of MANF mRNA in an m6A-dependent manner. **A** RIP assay assessing the binding of YTHDF family on MANF mRNA in ACHN cell line. Enrichment levels were normalized to IgG group. **B** RIP assay assessing the binding of YTHDF family on MANF mRNA in 786-O cell line. Enrichment levels were normalized to IgG group. **C** Actinomycin D assay assessing the stability of MANF mRNA in ACHN cell lines with/without YTHDF1 knockdown. Levels were normalized to siNC group, 0 h. **D** Actinomycin D assay assessing the stability of MANF mRNA in ACHN cell lines with/without YTHDF2 knockdown. Levels were normalized to siNC group, 0 h. **E** Actinomycin D assay assessing the stability of MANF mRNA in ACHN cell lines with/without YTHDF3 knockdown. Levels were normalized to siNC group, 0 h. **F** Western blot detecting the expression of MANF in siNC and siYTHDF2 cell lines (upper: ACHN, lower: 786-O). **G** RIP assay assessing the binding between YTHDF2 and MANF mRNA in ACHN cell line with/without ALKBH5 knockout. Enrichment levels were normalized to IgG group. **H** RIP assay assessing the binding between YTHDF2 and MANF mRNA in 786-O cell line with/without ALKBH5 knockout. Enrichment levels were normalized to IgG group. **I** schematic diagram of MANF mRNA m6A sites containing domain that could be recognized by YTHDF2. **J** Luciferase reporter assay of MANF different groups of point mutation in 293 cell lines with NC, ALKBH5 KO or YTHDF2 knockdown. Levels were normalized to NC/Vector group.

Several m6A readers execute the regulatory function on mRNAs, and among these readers, the YT521-B homology domain family (YTHDF) proteins are one of the most important families that control the splicing, translation, and decay of mRNA in a m6A-dependent manner. Thus, we further investigated whether one or more of these proteins recognizes the m6A modification on MANF mRNA and regulates the stability change. RIP was performed using a flag antibody in three different YTHDF-flag-overexpressing RCC cells. YTHDF2 was the most consistent protein that interacted with MANF mRNA (Fig. 5A, B), and knockdown of YTHDF2, but not YTHDF1 and YTHDF3, significantly elevated MANF mRNA expression (Fig. 5C–E). Also, the protein expression of MANF was consistently elevated after YTHDF2 knockdown (Fig. 5F). Moreover, ALKBH5 knockdown resulted in increased enrichment of MANF in theYTHDF2-flag pull-down group (Fig. 5G, H). Regarding m6A site-specific analysis, YTHDF2 may potentially recognize sequences in the m6A sites in group 1 and group 3 (Fig. 5I). Luciferase reporter assay with different m6A site mutation confirmed that YTHDF2 primarily bound to group 3 (Fig. 5J). These results demonstrated that YTHDF2 destabilizes MANF mRNA in an m6A-dependent manner.

### ER stress triggered by ALKBH5/MANF loss is exacerbated by VHL loss in RCC cells

**Fig. 6:**
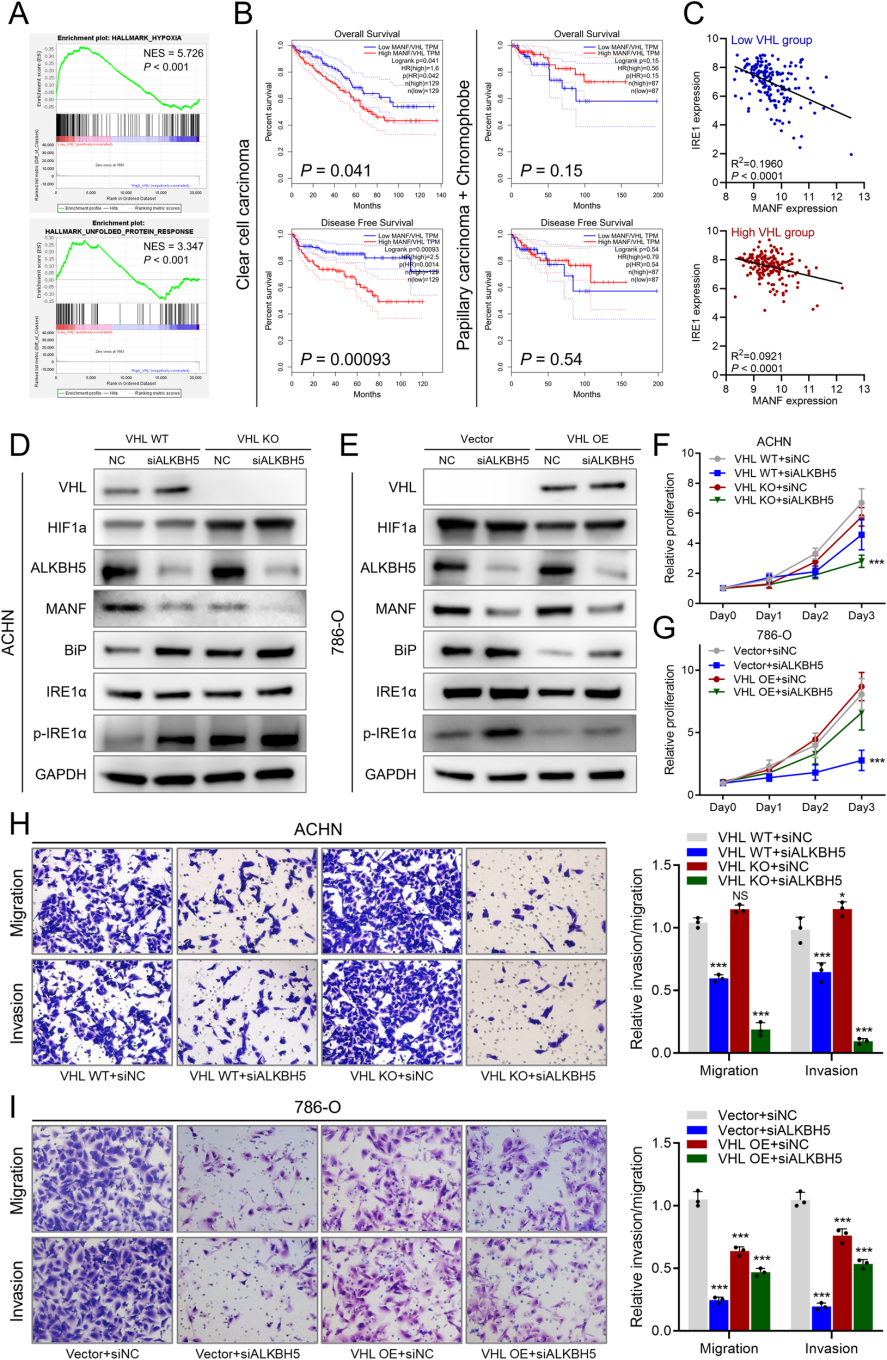
ER stress triggered by ALKBH5/MANF loss is exacerbated by VHL loss in RCC cells. **A** GSEA analysis of pathway activation status in high/low VHL expression groups of TCGA ccRCC patients. Upper: hypoxia pathway; lower: unfolded protein response. NES: normalized enrichment score. **B** Survival analysis of the high/low MANF groups normalized by VHL expression. Data was from TCGA patients. Left: TCGA ccRCC patient dataset; right: TCGA papillary and chromophobe dataset. **C** Correlation analysis of MANF and IRE1α in TCGA ccRCC dataset. Upper: low VHL expression group; lower: high VHL expression group. **D** Western blot detecting MANF and the ER stress effector proteins in ACHN cell line with NC/siALKBH5 with/without VHL knockout. **E** Western blot detecting MANF and the ER stress effector proteins in 786-O cell line with NC/siALKBH5 with/without VHL knockout. **F** CCK8 assay of ACHN cell line with NC/siALKBH5 with/without VHL knockout. Proliferation rates were normalized to day 0. **G** CCK8 assay of 786-O cell line with NC/siALKBH5 with/without VHL overexpression. Proliferation rates were normalized to day 0. **H** Transwell assay (migration and invasion) of ACHN cell line with NC/siALKBH5 with/without VHL knockout. Representative pictures shown in left and quantification data shown in right. **I** Transwell assay (migration and invasion) of 786-O cell line with NC/siALKBH5 with/without VHL overexpression. Representative pictures shown in left and quantification data shown in right.

Based on our observation, ALKBH5 and MANF alterations caused more dramatic changes in 786-O than in ACHN (Fig. 4B–I, Fig. 1D, E and J–M). This indicated that compared to 786-O cells, ACHN cells were less vulnerable to ALKBH5 knockout as indicated by suppressed cell growth and invasiveness alterations. Based on genetic aspect, the major difference between 786-O and ACHN cells is that the former cells harbor a VHL mutation [19]. A previous study has confirmed that VHL-mutant kidney cells are more prone to UPR and the resultant ER stress damage [6]. Given that HIF is a powerful transcriptional factor, we speculated that VHL mutation causing HIF overactivation will result in potential UPR, which was amplified by ALKBH5 and MANF alterations. This was confirmed with gene set enrichment analysis, showing low VHL expression group of TCGA ccRCC patients had activated hypoxia and UPR pathway (Fig. 6A). Moreover, after normalized by VHL expression, MANF expression correlated with the OS and DFS of ccRCC patients, which was not seen in papillary carcinoma and chromophobe (Fig. 6B). Also, in ccRCC dataset, the expression of IRE1α had a tighter relation with MANF in the low VHL group (R^2^: 0.1960 in low VHL group vs. 0.0921 in high VHL group, Fig. 6C). To further validate this, we established a VHL knockout ACHN cell line and a VHL overexpression 786-O cell line, and reevaluated the effect of ALKBH5. Simultaneous knockout of VHL and knockdown of ALKBH5 in ACHN significantly enhanced the ER stress caused by ALKBH5 knockdown alone as indicated by changes in the protein levels of ER stress markers, as well as proliferation and invasiveness phenotypes (Fig. 6D, F and H). Also, overexpression of VHL in 786-O ameliorated the damage caused by ALKBH5 knockdown (Fig. 6E, G and I). These results indicated that VHL loss could exacerbate the ER stress damage caused by ALKBH5.

### RCC cells have an intact regulatory ALKBH5-MANF-ER stress axis

**Fig. 7:**
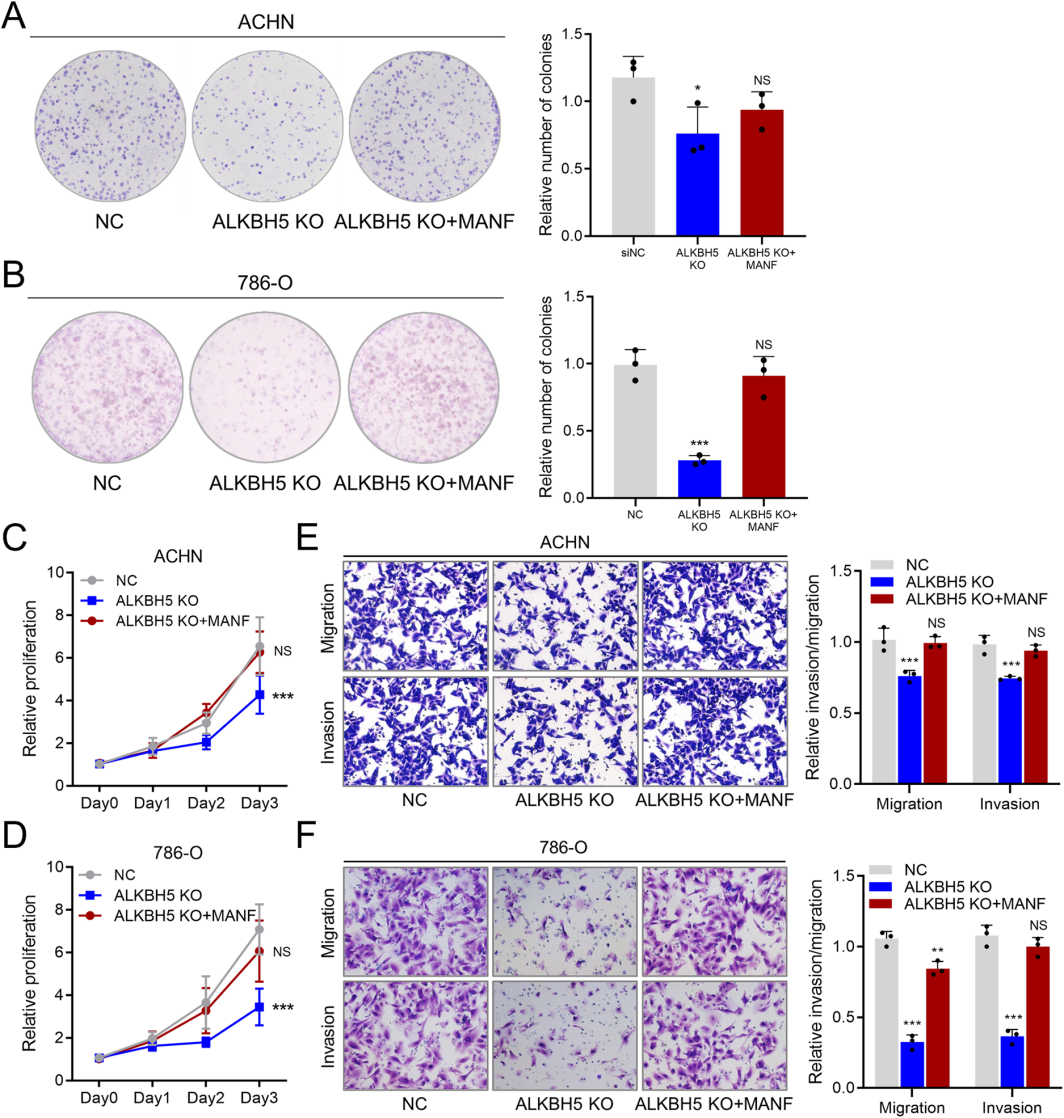
RCC cells have an intact regulatory ALKBH5-MANF-ER stress axis. **A** Colony forming assay of NC, ALKBH5 KO and ALKBH5 KO + MANF in ACHN cell line. Representative pictures shown in left and quantification data shown in right. **B** Colony forming assay of NC, ALKBH5 KO and ALKBH5 KO + MANF in 786-O cell line. Representative pictures shown in left and quantification data shown in right. **C** CCK8 assay of NC, ALKBH5 KO and ALKBH5 KO + MANF in ACHN cell line. Proliferation rates were normalized to day 0. **D** CCK8 assay of NC, ALKBH5 KO and ALKBH5 KO + MANF in 786-O cell line. Proliferation rates were normalized to day 0. **E** Transwell assay (migration and invasion) of NC, ALKBH5 KO and ALKBH5 KO + MANF in ACHN cell line. Representative pictures shown in left and quantification data shown in right. **F** Transwell assay (migration and invasion) of NC, ALKBH5 KO and ALKBH5 KO + MANF in 786-O cell line. Representative pictures shown in left and quantification data shown in right.

To validate the integrity of the ALKBH5-MANF-ER stress axis, rescue experiments were performed. Knockout of ALKBH5 significantly decreased the colony-forming ability and proliferation of RCC cells, and these phenotypes were rescued with reintroduction of MANF (Fig. 7A–D). Similar results were observed in transwell experiments (Fig. 7E, F). These data demonstrated that an intact ALKBH5-MANF-ER stress axis exists in RCC cells, controlling the ER status and overall cancer cell aggressiveness.

### ALKBH5 controls MANF and ER stress of RCC tumors in vivo

**Fig. 8:**
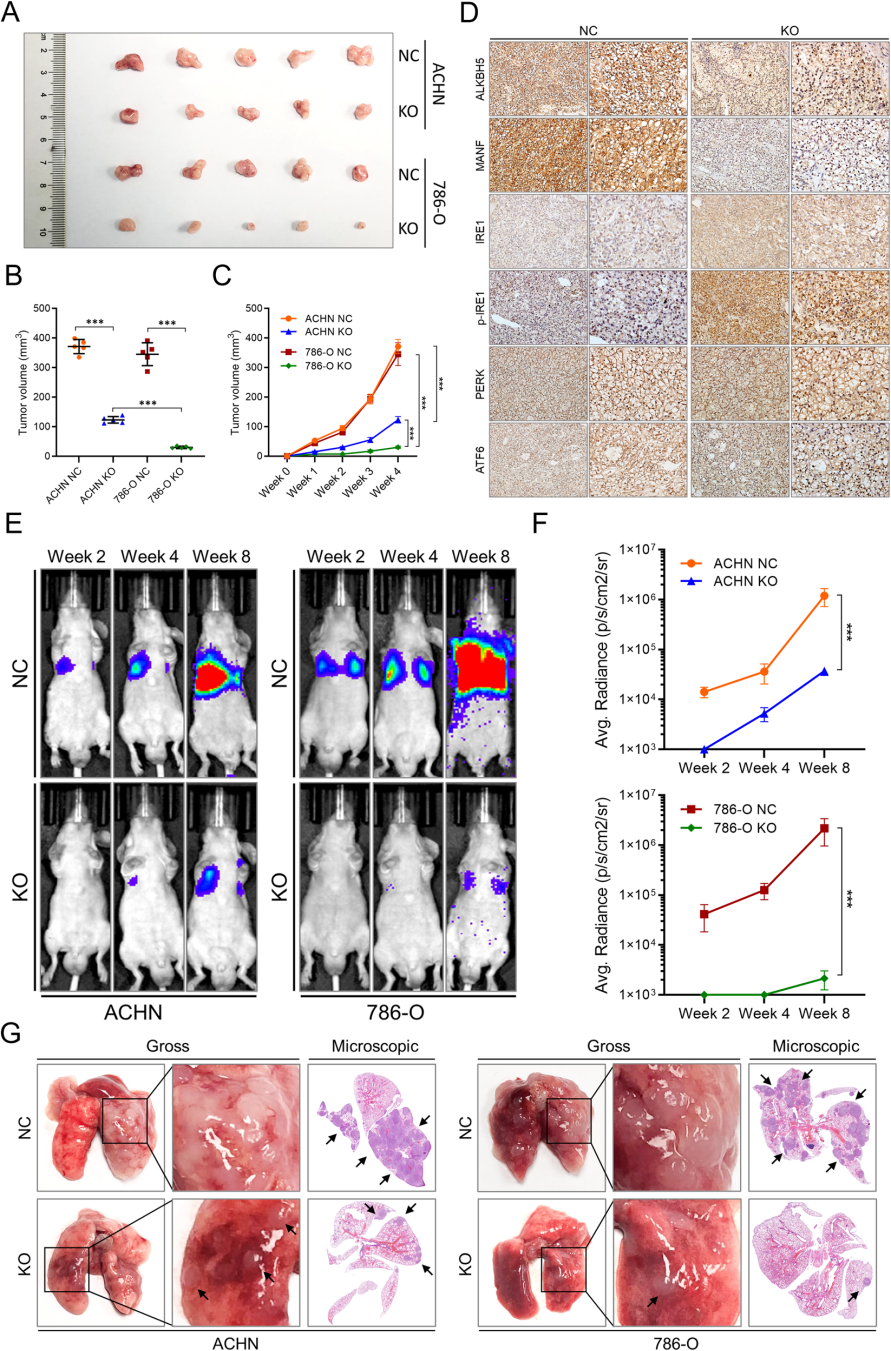
ALKBH5 controls MANF and ER stress of RCC tumors in vivo. **A** The representative picture of subcutaneous tumors of NC or ALKBH5 KO in ACHN and 786-O cell lines. **B** Final volumes of subcutaneous tumors of NC or ALKBH5 KO in ACHN and 786-O cell lines at week 4. **C** The volume change of subcutaneous tumors of NC or ALKBH5 KO in ACHN and 786-O cell lines from week 0 to week 4. **D** IHC staining of MANF and ER stress effectors in NC or ALKBH5 KO ACHN subcutaneous tumors. **E** Representative pictures of mouse lung metastasis model of NC or ALKBH5 KO in ACHN and 786-O cell lines. Tumor signals were detected with luciferase live imaging. The luminous area around lung indicated the intensity of metastasis. **F** The quantification of the mouse lung metastasis model of NC or ALKBH5 KO in ACHN and 786-O cell lines. **G** Representative pictures of mouse lung metastasis model of NC or ALKBH5 KO in ACHN and 786-O cell lines. Gross lung samples and their microscopic inspections were shown. Black arrows indicated metastatic nodules.

To further evaluate the role of ALKBH5 in vivo, animal experiments with control and ALKBH5 knockout cells were performed. In a subcutaneous tumor formation model, ALKBH5 knockout significantly restricted the growth of tumors (Fig. 8A–C). Moreover, IHC staining of tumors demonstrated decreased MANF, elevated IRE1α after ALKBH5 knockout (Fig. 8D). In the tail vein injection lung metastasis model, knockout of ALKBH5 caused less pulmonary colonization as indicated by in vivo imaging and ex vivo biopsy inspection (Fig. 8E–G). Further, mice in the control group experienced more intense cachexia as indicated by rapid weight loss. These results demonstrated that ALKBH5 exerts similar function in vivo by regulating RCC cell ER stress through MANF.

## Discussion

MANF was originally identified in the rat ventral mesencephalic cell line in 2003 [7], and later studies have reported that MANF has a neuroprotective effect in Parkinson’s disease and ethanol-induced neurodegeneration [12, 16]. Moreover, it has been further confirmed that MANF maintain neural cells survival by interacts with IRE1α and competes with BiP for binding to IRE1α [10, 11]. MANF physiologically has higher affinity to IRE1α, thus making IRE1α the initial responder [11]. The possible mechanism for MANF inhibiting ER stress is that: upon ER stress is triggered, BiP disassociates from IRE1α. Normally, IRE1α will go through oligomerization and mediate the subsequent ER stress reaction. However, when high concentration of MANF is presented, it directly binds to the luminal domain of IRE1α and prevents its oligomerization and phosphorylation, thus ameliorating ER stress [11]. Similarly, the present study also demonstrated increasing phosphorylated IRE1α and BiP when knocking down MANF in renal tumor cells. Furthermore, no significant changes were observed in the other two branches such as PERK and ATF6 following treatment of renal tumor cells with MANF siRNA. Thus, we speculated that MANF interacted with IRE1α and played an pro-survival activity in renal tumor progress through protecting ER stress and alleviates UPR. This study was the first to show that MANF promoted tumor growth in renal cell carcinoma by reducing UPR through ALKBH5 epigenetic regulation. Further research is needed to determine if ALKBH5 could be a potential therapeutic target.

ALKBH5 is a well-established regulator of m6A homeostasis, and it has been studied extensively in different types of cancers. Depending on the cancer type, ALKBH5 has distinct behaviors, ranging from oncogene to tumor suppressor [20]. For example, in glioblastoma, ALKBH5 maintains tumorigenicity of cancer stem-like cells by inducing the expression of FOXM1 [13]. However, in pancreatic cancer, ALKBH5 regulates the expression of Wnt inhibitory factor 1 (WIF-1) and inhibits the downstream Wnt signaling pathway [21]. The duality of ALKBH5 may be explained by the fact that ALKBH5 regulates various genes through its m6A eraser function. The behavior of ALKBH5 is even more complex in RCC as there are several pathological subtypes such as clear cell, papillary, and chromophobe cell carcinoma. Each subtype demonstrates distinct molecular and genomic characteristics, which contribute to the different responses to ALKBH5 [22]. A previous study has reported that ALKBH5 regulates the expression of aurora kinase B (AURKB) through its m6A eraser function in renal tumor, but a detailed mechanism was missing [23]. Our study delved into a novel mechanism through which ALKBH5 facilitated the transcription of MANF in clear cell renal cell carcinoma via a YTHDF2-dependent regulatory manner.

Further experiments showed that ALKBH5 demethylated the m6A sites on MANF mRNA, including those located on the 3’-UTR, and subsequently influenced the stability of MANF mRNA through a YTHDF2-dependent manner. These is consistent with the previous findings that the majority of functional m6A locates on the 3’-UTR near the stop codon, and YTHDF2 mainly recognizes these 3’-UTR m6A and controls the decay of mRNA [24]. MANF has been suggested to interact with IRE1α and promotes neuronal survival upon ER stress [16]. In the present study, loss of MANF induced unresolved ER stress in RCC cells, resulting in cell death and decreased invasiveness, which confirmed previous knowledge. Moreover, our phenotypic experiments demonstrated that ALKBH5 knockout even had a stronger impact on VHL-deficient 786-O RCC cells, compared to VHL wild-type ACHN cells [19]. These findings suggested that targeting ALKBH5 might be a promising therapeutic strategy for RCC cases with VHL loss. In mouse renal cell tumor models, the role of ALKBH5 in promoting tumor growth through the activation of tumor cells UPR and its relationship with MANF were investigated. Taken together, our data indicated that elevated levels of the m6A demethylase ALKBH5 in renal cell carcinoma may lead to increased expression of downstream MANF, activating tumor cell UPR, and resulting in cells proliferation and invasion.

The *VHL*/HIF axis in clear cell renal carcinoma always plays a key role. *VHL* mutation is up to 90% in sporadic ccRCC cases [25]. Using data from the TCGA database, we conducted bioinformatics analysis and found that hypoxia and UPR genes were significantly enriched in clear cell renal cell carcinoma. MANF low-expressions predicted favor prognosis specifically in ccRCC when normalized by *VHL*. To further elucidate wether ALKBH5/MANF related UPR was related to VHL in RCC, two representative cell lines were selected as models. ACHN is characterized as a VHL wild-type, while 786-O harbors VHL mutation [19]. Either ALKBH5 knockout or MANF ablation had a more noticeable effect on VHL inactivation 786-O, suggested tumor cell ER stress protective effects and mechanisms of MANF was affected by VHL status. A previous study has reported that VHL mutation induces an aversive ER stress activation status in precancerous kidney cells [6]. Together, we have identified a mechanism in VHL inactivated renal tumor cells that involves epigenetic regulation of MANF to adapt to ER stress caused by rapid cell growth. Further research is needed to fully understand this process.

## Conclusion

**Fig. 9:**
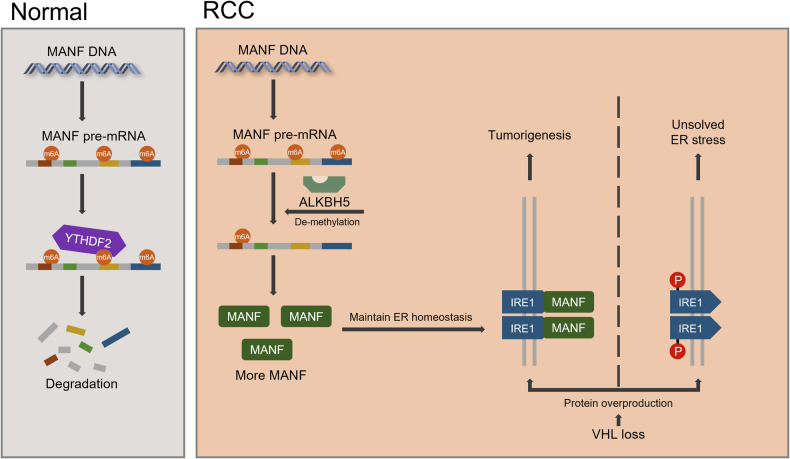
Schematic diagram of the mechanism of ALKBH5, VHL and ER stress in RCC. Schematic diagram of the mechanism of ALKBH5, VHL and ER stress in RCC. Created with BioRender.com.

The findings of this study indicated that the m6A eraser ALKBH5 upregulated *MANF* in a YTHDF2-dependent fashion, thereby conferring protection against ER stress-induced cell death in renal cell carcinoma. Our data provided a new understanding and potential targets for RCC therapy (Fig. 9).

## Supplementary information

Supplemental Figures

Table S1

Gels and Blots images

## Data Availability

For all data requests, please contact the corresponding author.
