# Supplementary material for: N6-methyladenosine-mediated upregulation of MANF promotes ER stress resistance in renal cell carcinoma
Source: Cell Death Dis. 2025 Jul 3;16(1):486. doi: 10.1038/s41419-025-07798-4 (PMC12222710; doi:10.1038/s41419-025-07798-4)
Supplement: Supplementary file 1 — Supplemental Figures [file 41419_2025_7798_MOESM1_ESM.docx]

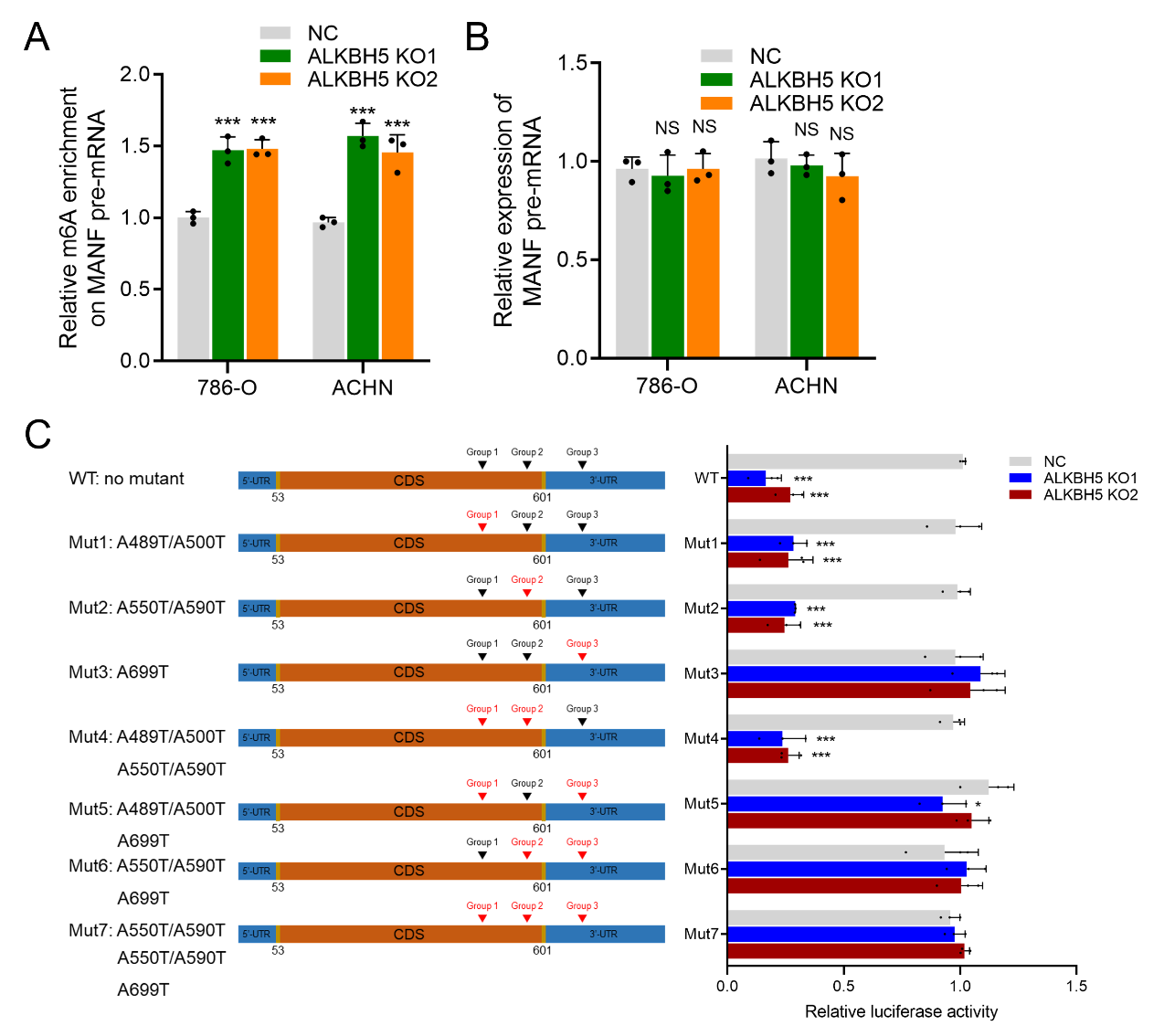


**Figure S1. ALKBH5 modulates the m6A abundance on MANF mRNA 3’-UTR**

**A**. m6A pull-down assay assessing the m6A abundance of MANF pre-mRNA in ACHN and 786-O cell lines with NC or ALKBH5 KO. Enrichment levels were normalized to NC group. **B**. qRT-PCR detection of MANF pre-mRNA expression level in in ACHN and 786-O cell lines with NC or ALKBH5 KO. Enrichment levels were normalized to NC group. **C**. Left: schematic diagram of MANF mRNA structure and the different combination point mutation(s) of three groups of m6A sites. Right: Luciferase reporter assay different combination point mutation(s) of three groups of m6A sites on MANF mRNA in 293 cell lines with NC or ALKBH5 KO. Levels were normalized to NC/MANF-WT group.


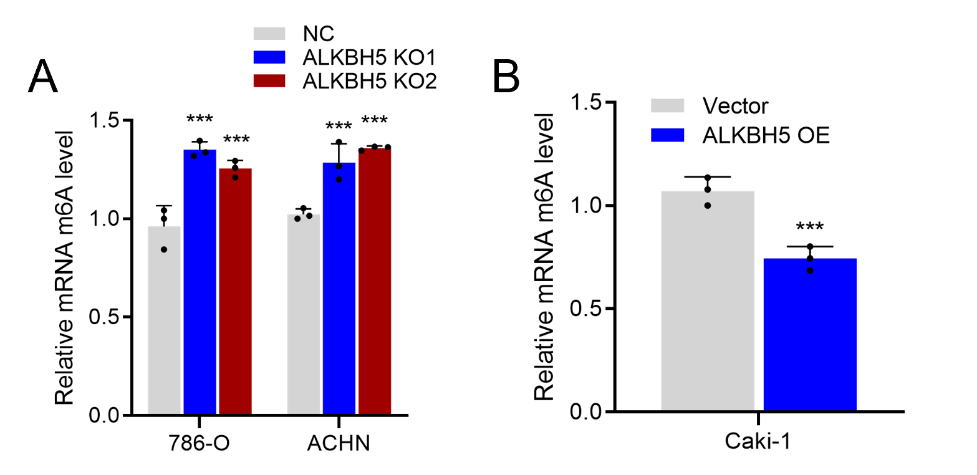


**Figure S2. m6A levels after ALKBH5 knockdown or overexpression**

**A.** The overall m6A level change of NC, ALKBH5 KO1 and ALKBH5 KO2 in ACHN and 786-O cell lines. **B**. The overall m6A level change of Vector and ALKBH5 OE in Caki-1 cell line.
