# Supplementary material for: N6-methyladenosine-mediated upregulation of MANF promotes ER stress resistance in renal cell carcinoma
Source: Cell Death Dis. 2025 Jul 3;16(1):486. doi: 10.1038/s41419-025-07798-4 (PMC12222710; doi:10.1038/s41419-025-07798-4)

Fig. 1D

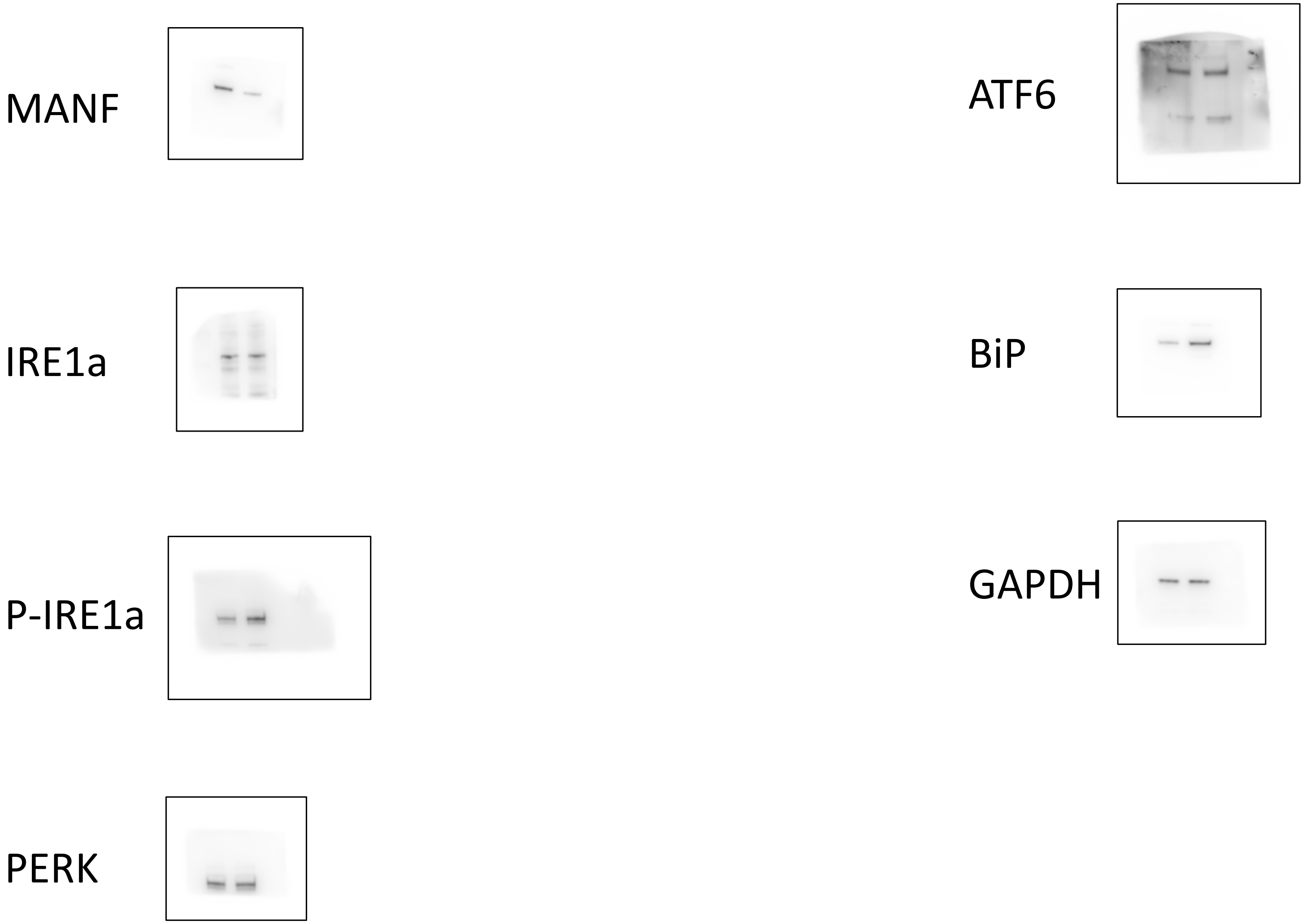

Fig. 1E

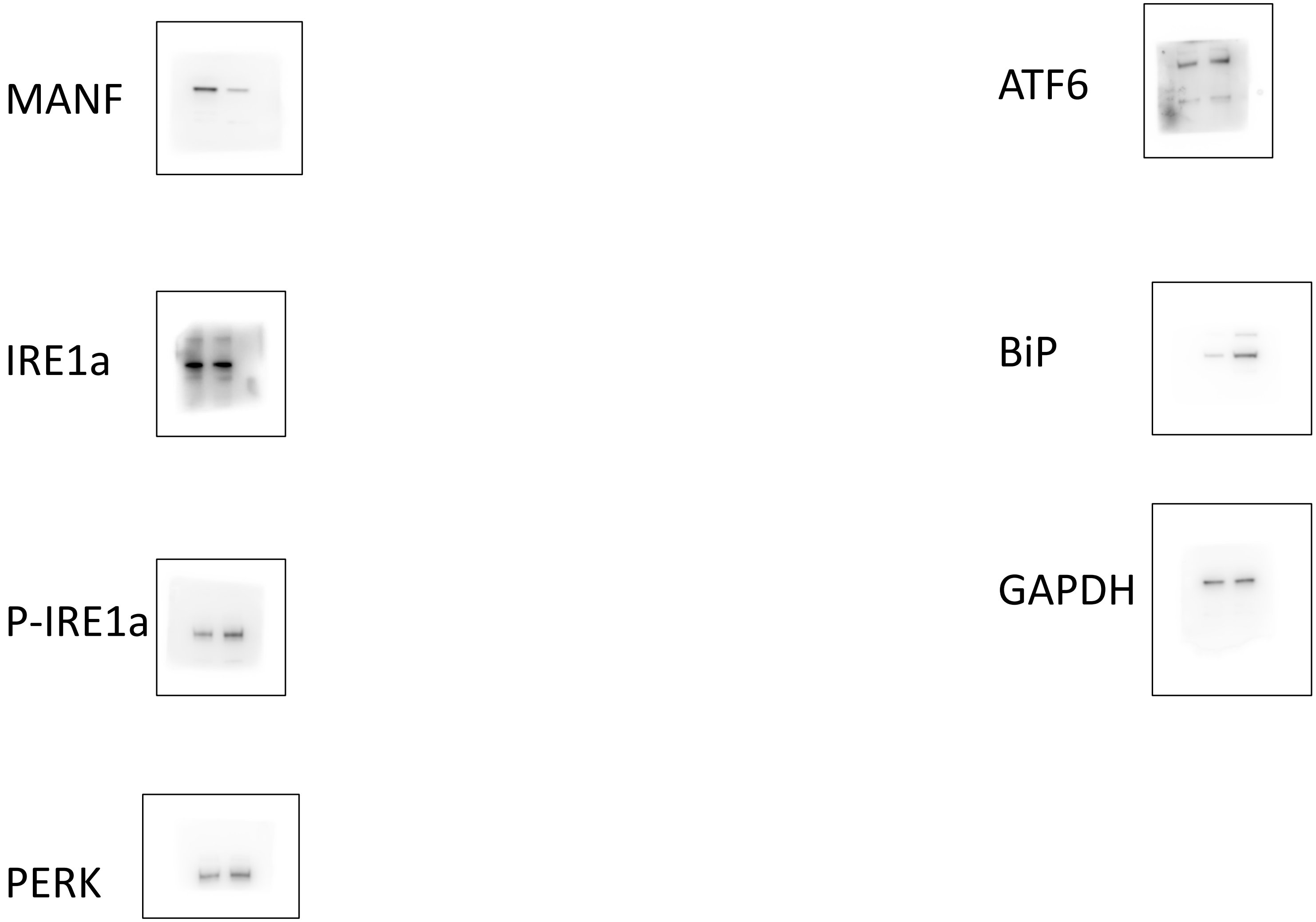

Fig. 4J

ALKBH5

GAPDH

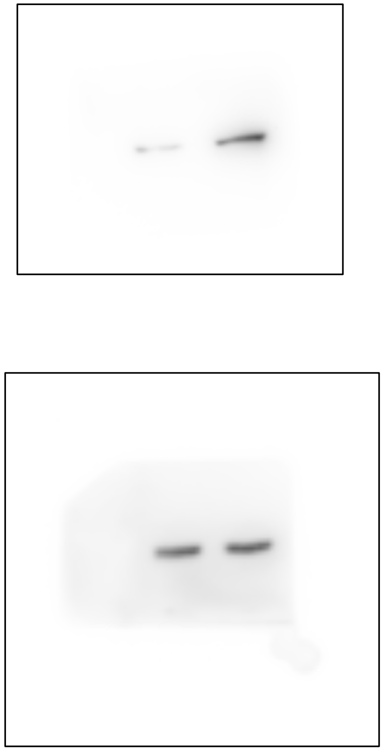

Fig. 4A

ACHN

ALKBH5

GAPDH

786-O

ALKBH5

GAPDH

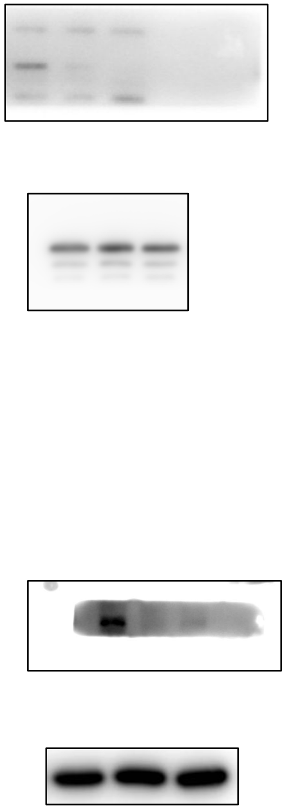

Fig. 5F

786-O

MANF

GAPDH

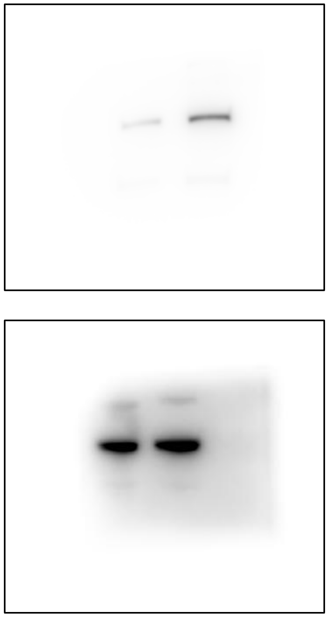

ACHN

MANF

GAPDH

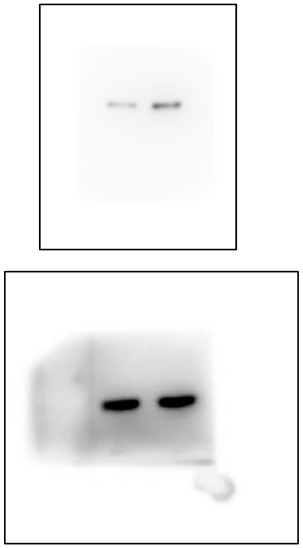

Fig. 6D

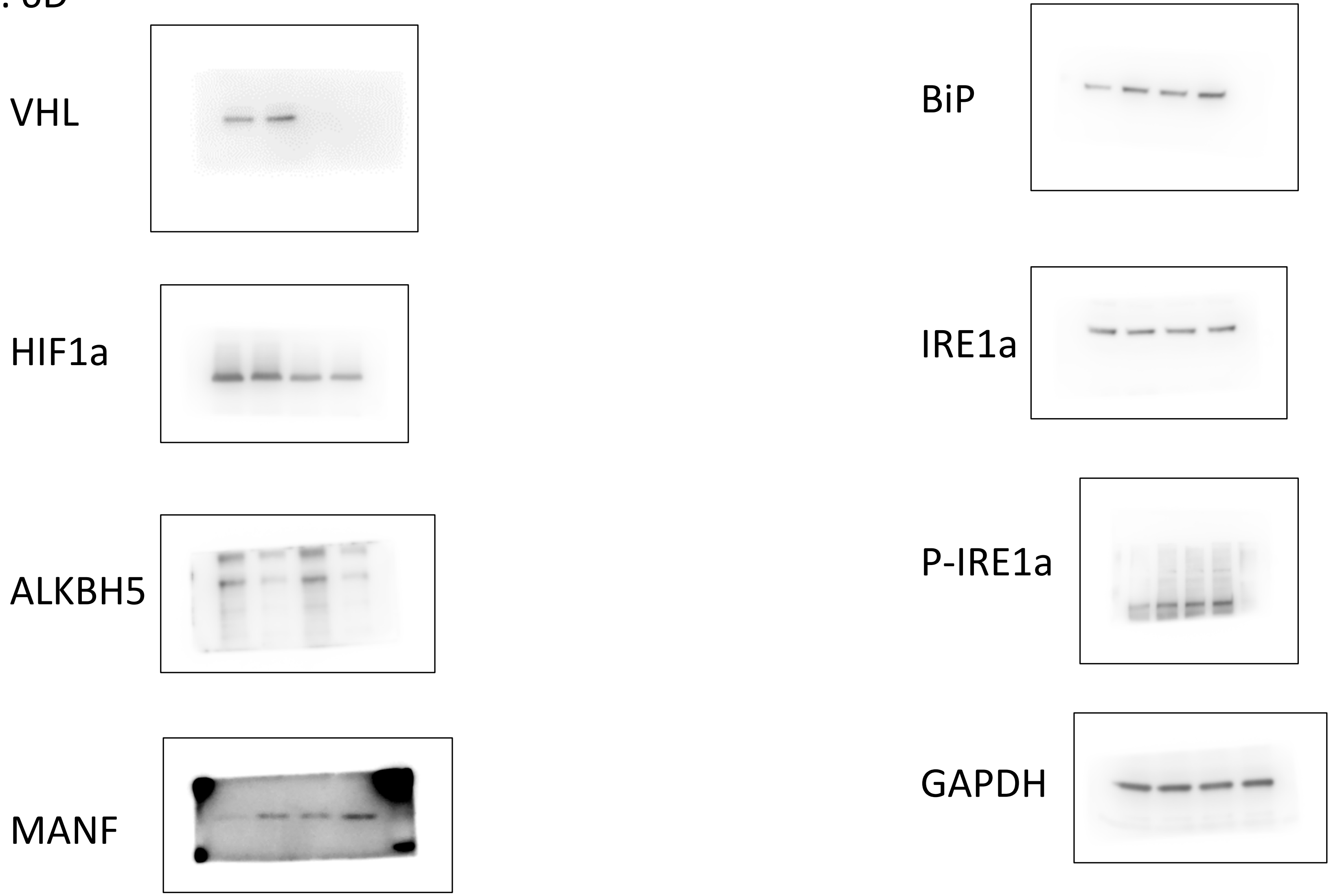

Fig. 6E

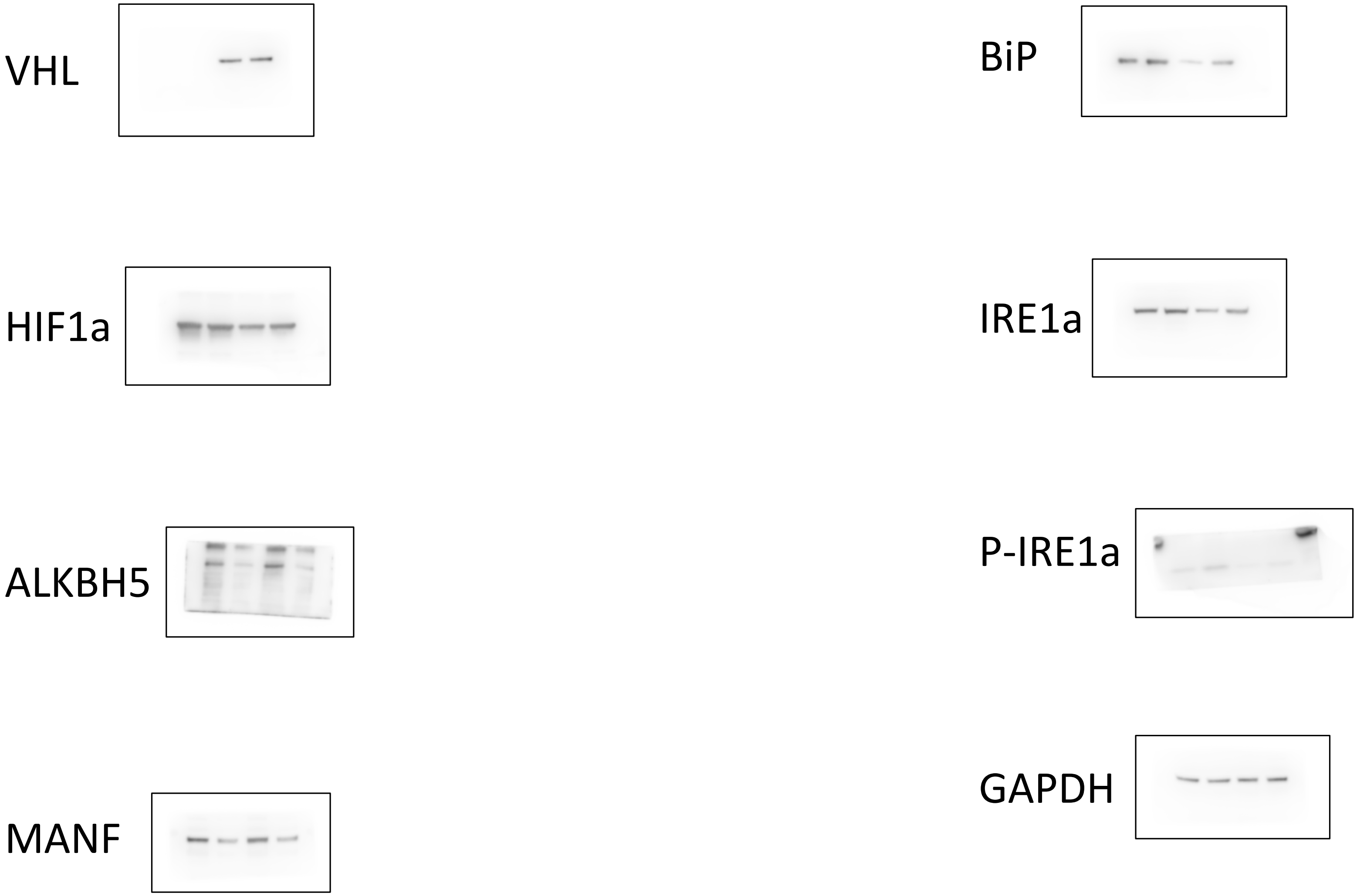

Supplement: Supplementary file 3 — Gels and Blots images [file 41419_2025_7798_MOESM3_ESM.pdf]
